# Proposal of a new visual analogue scale to describe the extent of lymphadenectomy in right-sided colectomy for cancer—a prospective observational study

**DOI:** 10.1007/s10151-025-03182-8

**Published:** 2025-09-02

**Authors:** F. Pfeffer, P. Kalgraff, K. B. Lygre, B. S. Nedrebø, H. M. Forsmo

**Affiliations:** 1 Department of Gastrointestinal Surgery, Haukeland University Hospital, 5021 Bergen, Norway; 2 Department of Clinical Medicine, University of Bergen, Bergen, Norway

**Keywords:** Right-sided colon cancer, Lymphadenectomy, Visual analogue scale, Surgical quality assessment, Mesenteric lymph node dissection, Specimen evaluation

## Abstract

**Background:**

Lymphadenectomy in right-sided colon cancer lacks standardized reporting. The aim was to develop a visual analogue scale (VAS) based on mesenteric vessels to describe the extent of lymphadenectomy.

**Methods:**

We included patients undergoing surgery for right-sided colon cancer from January 2021 to September 2024. Data were collected via a web-based database. Immediately after surgery, surgeons recorded the VAS score, vascular visualization, and specimen quality.

**Results:**

Data from 155 patients were analyzed. Median age was 74 (IQR: 68–80), with 53% female. The median VAS score was 8.2 (IQR: 7.8–8.9). The superior mesenteric vein (SMV) was visualized in 84% of cases, with a median VAS score of 8.4 (IQR: 8.0–9.2) for visualized and 7.0 (IQR: 6.8–7.5) for non-visualized (*p* < 0.001). The gastrocolic trunk of Henle (GTH) was visualized in 51%, with a median VAS score of 8.7 (IQR: 8.3–9.7) for visualized and 7.9 (IQR: 7.3–8.0) for non-visualized (*p* < 0.001). Specimen quality was Type 0 (best) in 54% (VAS score 8.6, IQR: 8.2–9.5), Type I in 37% (VAS score 7.9, IQR: 7.3–8.0), and Type II in 6% (VAS score 6.9, IQR: 6.5–7.9; *p* < 0.001). A positive correlation between VAS score and lymph node count was found (*r* = 0.43, *p* < 0.001).

**Conclusions:**

The VAS score is a reliable and feasible method to describe lymphadenectomy in right-sided colon cancer. Unlike categorical classifications, the VAS score is based on anatomical landmarks and does not depend on consensus definitions. It reflects the visualization of vascular structures and correlates with specimen quality and lymph node yield.

**Clinical trial:**

ClinicalTrials.gov Identifier NCT06329102 (registered on March 24, 2024).

**Article type:**

Prospective observational study.

**Supplementary Information:**

The online version contains supplementary material available at 10.1007/s10151-025-03182-8.

## Introduction

Right- and left-sided colon cancer evolves from different embryological origins with different molecular profiles and vascular anatomies. The vascular anatomy of the right colon is more complex and involves multiple branches from both the superior mesenteric vein and artery [1, 2]. Due to the complex relationship between the vessels to the right colon, central lymphadenectomy in right colon cancer is more challenging than for left-sided cancers. Over the past decade, there has been an increasing focus on the management of right-sided colon cancer. The extent of lymphadenectomy and the integrity of the mesocolon are crucial to improve colon cancer surgery. Central lymphadenectomy in right-sided colon cancer involves dissection along the superior mesenteric axis. Two sets of terms describe lymph node harvest and dissection along embryological planes, but there is no clear consensus regarding the extent and the terminology of lymphadenectomy. The Japanese classification refers to dissection of central lymph node stations (D3) [3] and the European approach defines complete mesocolic excision (CME) as dissection between embryological planes with a central vascular tie [4].

Radical right-sided colon cancer surgery, particularly complete mesocolic excision (CME), has been associated with improved survival outcomes. However, variations in definitions and consensus regarding the extent of lymphadenectomy contribute to limited evidence.

A Japanese study demonstrated that D3 lymph node dissection in patients with pT3 and pT4 colon cancer was associated with a significant survival advantage compared to D2 lymph node dissection [5]. Research led by Bertelsen indicated that CME reduces cancer recurrence risk and enhances long-term survival rates [6]. In the RESECTAT trial, an unplanned exploratory analysis suggested that CME was associated with improved disease-free survival compared to standard surgery for stage III cancer [7]. Additionally, the RELARC study found that among stage III patients, the CME group exhibited a nearly 10 percentage point higher disease-free survival rate [8].

These findings underscore the potential of radical right-sided colon cancer surgery to improve outcomes, especially in advanced stages. Nevertheless, standardized definitions and further research are essential to fully understand the benefits and optimal application of these surgical techniques. In Norway, due to concerns about major complications, national guidelines recommend dissection and vascular ligation along the right side of the superior mesenteric vein (SMV). The term “complete D2” (cD2) is introduced and defined as dissection along the right border of the SMV. Vascular ligation of the ileocolic artery and vein is performed to the right of the SMV. The superior mesenteric vein is typically visualized from the lateral side. However, the interpretation of its definition can vary among surgeons. The “surgical trunk” of the specimen is not always preserved. In case of suspicious central lymph nodes on preoperative computed tomography, a D3 dissection is recommended. However, there is no national consensus about the extent of the D3 dissection. The most widely accepted definition involves dissection along the left border of the SMV, though definitions also include dissection to the left of the superior mesenteric artery (SMA) [9].

A recent systematic review identified significant variability in the reporting and definitions of radical right-sided colon cancer surgery with multiple combinations of definitions published [10]. The lack of consensus undermines assessment of the influence of the extent of lymphadenectomy on oncological outcome.

**Fig. 1:**
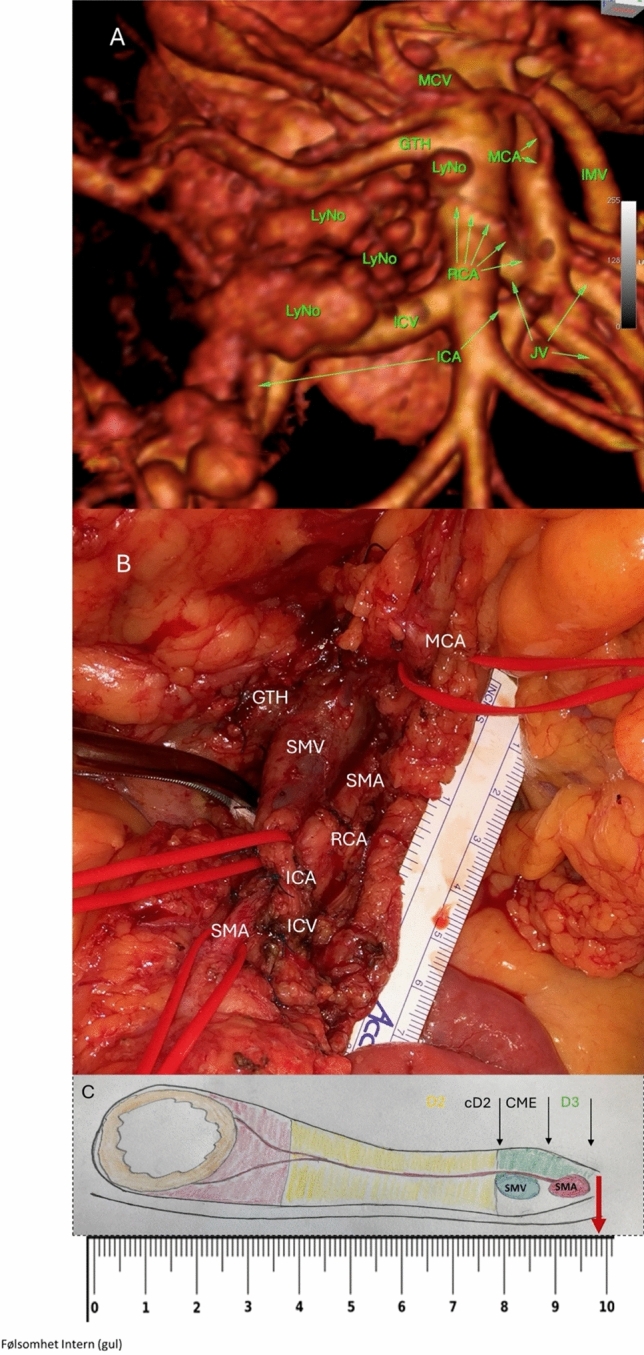
**A** Preoperative computed tomography (CT) with contrast, 1-mm slice thickness, and three-dimensional volume rendering Osirix reconstruction of the root of the midgut mesentery. **B** Same patient as in **A**, image acquired at surgery. **C** Schematic drawing of the mesenteric vessels superior mesenteric vein (SMV) and superior mesenteric artery (SMA) at the level of the ileocolic vessels combined with a visual analogue scale. Categorical lymph node dissection: D2, complete D2 (cD2), complete mesocolic excision (CME), and D3. Red arrow represents the extent of lymphadenectomy as shown in **B**. VAS score 9.8. Lymph node (LyN0), ileocolic vein (ICV), gastrocolic trunk of Henle (GTH), middle colic artery (MCA), right colic artery (RCA), superior mesenteric vein (SMV), and superior mesenteric artery (SMA)

Although the vascular anatomy of the right colon is complex, the superior mesenteric artery (SMA) is consistently positioned medial or dorso-medial to the superior mesenteric vein (SMV), from the branching of the ileocolic artery upwards. The venous drainage to the gastrocolic trunk of Henle (GTH) exhibits several variations but remains largely consistent [11]. The D3 area's inferior border is marked by the ileocolic vessels, while its cranial border is defined by the gastrocolic trunk of Henle. The consistent anatomical relationship between the SMV and SMA at the level of the ileocolic vessels served as the foundation for developing the visual analogue scale. A schematic diagram depicting the vascular anatomy was created and integrated into the scale (Fig. 1).

To address the lack of consensus and provide a more objective description of the extent of lymphadenectomy, the visual analogue scale based on vascular anatomy was introduced. This scale was then compared with the surgeon’s categorical classification, the visualization of mesenteric vessels, the quality of the specimen, and the lymph node harvest.

## Methods

In a prospective observational cohort study, we collected data from 155 patients scheduled for surgery for right-sided colon cancer at Haukeland University Hospital in Bergen, Norway, between January 2021 and September 2024. The study is reported in line with the Strengthening the Reporting of Observational studies in Epidemiology (STROBE) guidelines.

Throughout the study period, four surgeons with expertise in central lymphadenectomy, referred to as D3 surgeons, were involved. Three D3 surgeons, experienced in central lymphadenectomy since 2012, participated in a randomized trial comparing open D3 lymphadenectomy with laparoscopic complete mesocolic excision (CME) for right-sided colon cancer [12]. Two D3 surgeons (HMF, FP) developed the visual analogue scale. Figure 1 illustrates the application of the visual analogue scale (VAS) to quantify the extent of mesenteric lymphadenectomy in a single patient undergoing right-sided colon cancer surgery. The figure integrates a preoperative contrast-enhanced CT scan with 3D Osirix reconstruction of the midgut mesentery, an intraoperative photograph, and a schematic representation of the vascular anatomy at the ileocolic level. The red arrow highlights the dissection area, corresponding to a VAS score of 9.8. This score reflects an extensive lymphadenectomy approaching the D3 level, with clear identification of key vascular structures, including the superior mesenteric artery (SMA), superior mesenteric vein (SMV), and their associated branches. This example highlights the utility of the VAS as a precise, anatomy-based tool for assessing the extent of lymphadenectomy. Additional patient examples illustrating the application of the VAS are presented in Figure S1 within the supplementary appendix.

A prospective web-based database was established to evaluate the impact of surgical access and the extent of lymphadenectomy on postoperative outcomes.

The visual analogue scale (VAS) and paper form (Fig S1 in the supplementary appendix) were introduced to all colorectal surgeons. Standardization was achieved using intraoperative photodocumentation to illustrate the extent of lymphadenectomy corresponding to VAS scores. Junior surgeons were trained intraoperatively through direct supervision and guidance by experienced surgeons, ensuring consistent scoring across different experience levels. Younger colleagues were trained in vascular anatomy preoperatively by reviewing patients' CT scans. Axial and coronal reformats were used to assess the relationship between the ICA and the SMV, as well as to estimate the distance between the ICV and the GTH. While the GTH could often be approximated, identification of the MCA on preoperative CT was inconsistent and generally not reliable for surgical planning. As part of their specialization training, 18 other surgeons performed surgeries on right-sided colon cancer.

Immediately following the operation, the surgeon documented key details on a paper form regarding extent of lymphadenectomy (Fig. S1 in the supplementary appendix). This included the visual analogue scale (VAS) score (Fig. 1), visualization of the mesenteric vessels, and both the planned and achieved categorical extent of lymphadenectomy. The form also recorded data on blood loss, surgical access, and specimen quality. The categorical classification of lymphadenectomy extent was assigned according to national guidelines, with classifications of D2, cD2, and D3 [9].

The specimen quality was assessed with the classification system of Benz [13].

During 2021, eligible patients were also included in the open D3 arm of a randomized trial comparing open D3 lymphadenectomy versus laparoscopic CME in right-sided colon cancer [12]. After termination of this trial, the patients were operated on according to recommendations of the multidisciplinary team. Open surgery was recommended for locally advanced tumors or in case of contraindications for laparoscopy. Robotic-assisted laparoscopy was preferred when the system was available. A D2 lymphadenectomy was recommended for reoperations following endoscopically removed polyps or for very elderly and frail patients. In cases of suspected central lymph node metastasis, an open D3 lymphadenectomy was advised. During the study period, complete visualization of the SMV was established as the standard procedure for advanced tumors. Today, we refer to this procedure as CME.

All patients with histologically proven adenocarcinoma of the right-sided colon that gave written informed consent were included in the study. Patients with multiple tumors at other locations or ongoing oncological treatment of other malignant disease were excluded. Patients that could not give or refused consent were excluded.

All data were collected by a dedicated study nurse. Data source was the intraoperative paper form (Fig. S1 in the supplementary appendix) and the electronic patient chart. Pre- and postoperative data such as patient characteristics, pathological results, postoperative complications, and hospital stay were collected from the patient chart. Total hospital stay was defined as number of days of postoperative hospital stay and any additional hospitalization within the first 30 days after surgery.

The surgeon’s categorical classification was retrospectively adjusted based on the VAS score. To correlate the surgeon’s categorical classification with their VAS score, the following definitions were applied: D2 lymphadenectomy was classified as a VAS score ≤ 7.7, cD2 as VAS score > 7.7 to ≤ 8.0, CME as VAS score > 8.0 to ≤ 9.0, and D3 as VAS score > 9.0 (Fig. 1C). The classification of CME was introduced during the analysis. On the paper form, however, the surgeon could only select from the categories D2, cD2, or D3.

All patients gave written informed consent prior to operation. The study was approved by the Regional Committee of Ethics (REK 93628) and is in accordance with the “WMA Declaration of Helsinki-Ethical Principles for Medical Research Involving Human Subjects" [14].

### Statistics

The trial was preregistered (clinicaltrials.gov ID NCT06329102) before data analysis but after start of the study.

Continuous data were expressed as mean [median, lower and upper interquartile ranges (IQR)]. Intergroup comparisons were made using a Mann-Whitney U-test or one-way ANOVA for multiple groups. Tukey’s Honest Significant Difference (HSD) test was used as a post hoc analysis to assess the significance of differences between group means. Categorical data were expressed as numbers and percentages. We used the chi-square test as appropriate. With a density plot, the distribution of the VAS score and the total number of lymph nodes harvested was visualized. The association between VAS score and total number lymph nodes harvested was measured with linear regression and Pearson's correlation coefficient. Patients with missing data in at least one of the variables used for analysis were excluded. Data were collected in a web-based database, Ledidi Core, Ledidi AS, Oslo, Norway. All statistical analyses were performed directly in the dataset with inbuilt statistical tools.

## Results

From 21 January 2021 to 1 September 2024, 198 patients underwent scheduled operations for right-sided colon cancer at Haukeland University Hospital. One hundred fifty-five patients were included in the study and gave written informed consent. Thirty-seven patients were not included. The main reasons for exclusion were inability to consent due to dementia (*n* = 7); refusal to participate (*n* = 12); lack of study information provided for logistical reasons (*n* = 18); absence of the VAS score on the preoperative form submitted by the surgeon (*n* = 6). Patient characteristics, postoperative outcome, and tumor characteristics are presented in Table 1. In 133 (82%) of the surgeries, a D3 surgeon was part of the surgical team, and in 58% of cases, a D3 surgeon served as the primary surgeon. Additionally, 18 other surgeons performed surgeries for right-sided colon cancer. Thirteen of these surgeons operated on fewer than five patients, while five surgeons performed between six and ten surgeries.

**Table 1:** Patient characteristics, postoperative outcome, and tumor characteristics of the 155 patients included in the study

Characteristics	All patients (*n* = 155)
Gender	
Female	83 (53)
Male	72(47)
Age (years)	73 (74, 68–80)
BMI (kg/m^2^)	26 (26, 22–29)
ASA score	
1	6 (4)
2	77 (50)
3	69 (44)
4	1 (1)
Missing	2 (1)
Tumor location	
Cecum	69 (44)
Ascending colon	59 (38)
Hepatic flexure	15 (10)
Right transverse colon	12 (8)
Preoperative CEA (ng/ml)	27 (3, 2–7)
CT EMVI positive	42 (27)
Surgical access	
Open	56 (36)
Laparoscopic	49 (32)
Robotic	50 (32)
VAS score	8.3 (8.2, 7.8–8.9)
Bleeding (ml)	72 (20, 10–50)
Complications (Clavien-Dindo)	
Grade 0	96 (62)
Grade 1	22 (14)
Grade 2	17 (11)
Grade 3	18 (12)
Grade 4	1 (1)
Grade 5	1 (1)
Reoperation	12 (8)
Anastomotic leak	6 (4)
Hospital stay (days)	6 (4, 3–7)
Total hospital stay (days)	11 (9, 6–17)
pT-stage	
T0	6 (4)
T1	4 (3)
T2	17 (11)
T3	65 (42)
T4	59 (38)
Tx	2 (1)
pN-stage	
N0	91 (59)
N1	33 (21)
N2	26 (17)
N1c	1(1)
Total lymph node yield	23 (20, 15–28)
Adjuvant chemotherapy	
Yes	44 (28)
No	111 (72)

Data are given as *n* (%) or mean (median, interquartile ranges)

*BMI* body mass index, *ASA* American Society of Anesthesiologists, *CT EMVI* extramural venous invasion by CT, *VAS score* visual analogue scale score. Total hospital stay: Hospital stay within the first 30 days after surgery

Data were missing for the superior mesenteric vein (SMV) in five patients, for the gastrocolic trunk of Henle (GTH) in four patients, for the superior mesenteric artery (SMA) in four patients, and for specimen quality in six patients.

**Fig. 2:**
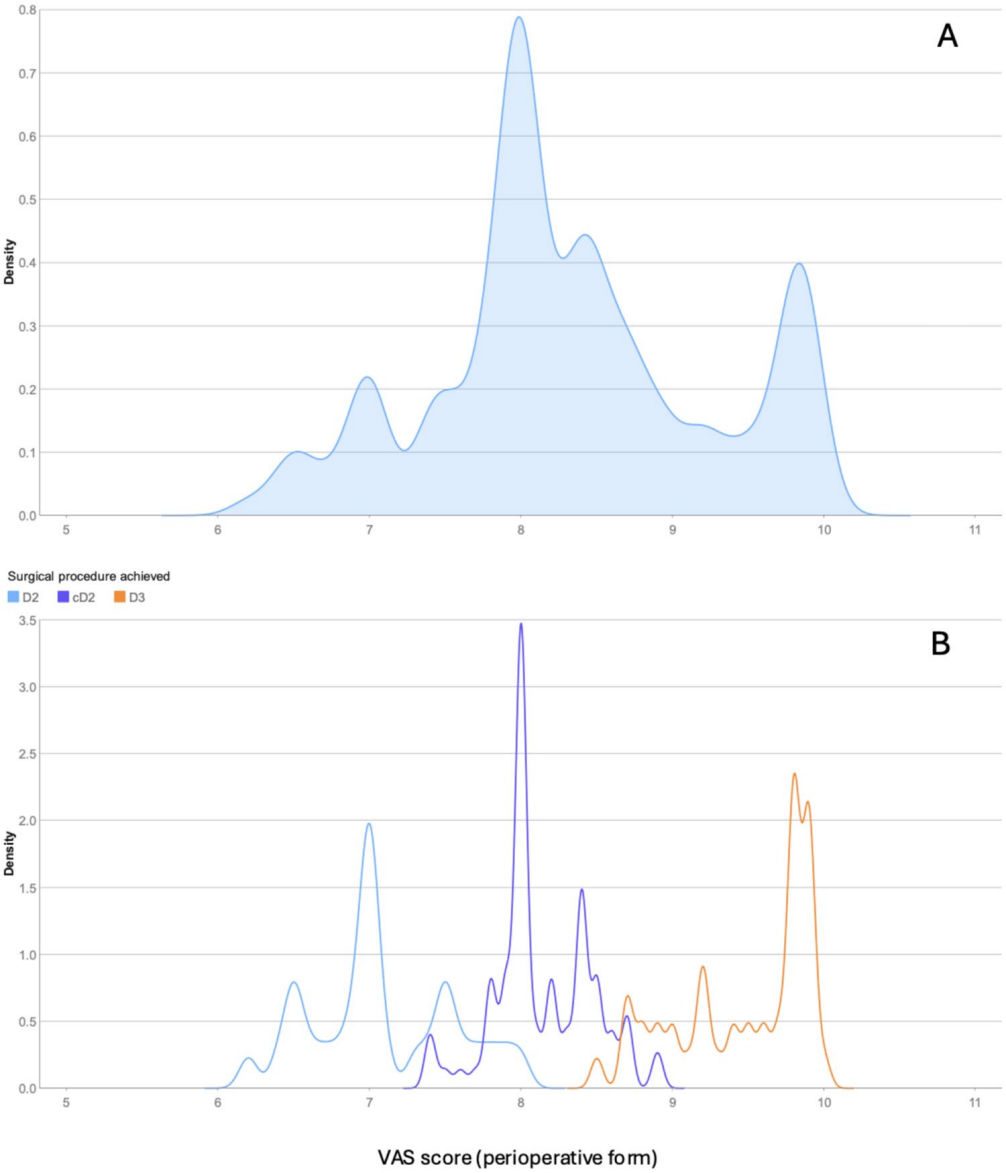
Density plot to illustrate the distribution of the VAS score and the total number of lymph nodes harvested. **A** All patients. **B** Patients grouped after the surgeon’s categorical classification

Mean VAS score for all patients was 8.3 (median 8.2, IQR 7.8–8.9) with a range from 6.2 to 10. The density plot (Fig. 2a) demonstrated a wide spread, reflecting the variability in the extent of lymphadenectomy. The plot displayed three peaks corresponding to the D2, cD2, and D3 categories of lymphadenectomy. The highest peak aligned with the right border of the superior mesenteric vein. The surgeon's categorical classification showed considerable overlap between the groups, along with some misclassification between cD2 and D3. (Fig. 2b).

The planned extent of lymphadenectomy differed significantly from the achieved result. The planned lymphadenectomy was achieved in 90% for planned D2, in 93% for planned cD2, and in 86% for planned D3 dissection (*p* < 0.001).

The surgeon classified the operations as follows: D2 in 29 patients, cD2 in 82 patients, and D3 in 44 patients. There was a significant variation in the VAS scores for each group: D2 had a VAS score of 7.1 (median 7.0, IQR 6.7–7.5), cD2 had a VAS score of 8.1 (median 8.0, IQR 8.0–8.4), and D3 had a VAS score of 9.5 (median 9.6, IQR 9.1–9.8), with a significant difference between the groups (*p* < 0.001).

Compared to the surgeon’s original classification, the corrected categorical classification of lymphadenectomy, based on the surgeon’s VAS score, showed a significant difference (*p* < 0.001). The extent of lymphadenectomy was categorized as follows: D2 in 32 patients (VAS ≤ 7.7), cD2 in 41 patients (VAS > 7.7 to ≤ 8.0), CME in 48 patients (VAS > 8.0 to ≤ 9.0), and D3 in 34 patients (VAS > 9.0).

The total lymph node yield in the corrected categorical classification, based on the surgeon’s VAS score, showed significant differences. The highest yield was observed in patients classified as D3, with a mean of 30 lymph nodes (median 30, IQR 23–36). The yields for the other groups were CME, 22 (median 19, IQR 14–27); cD2, 19 (median 16, IQR 13–22); and D2, 19 (median 16, IQR 13–22). The lymph node yield in the D3 classification was significantly higher than in the other groups (*p* < 0.001; Table 2).

**Table 2:** Total lymph node yield and classifications based on visual analogue scale (VAS) dependent on vascular anatomy

VAS classification	Number (%)	Total lymph node yield	*p*- value
D2	31 (21)	22 (19, 14–22)	*p* < 0.001^a^
cD2	41 (26)	19 (16, 13–22)	
CME	48 (31)	22 (19, 14–27)	
D3	34 (22)	30 (30, 23–36)	

Tukey’s post hoc test^b^	
D3 vs D2	*p* < 0.001
D3 vs cD2	*p* < 0.001
D3 vs CME	*p* < 0.001
CME vs cD2	*p* < 0.298
CME vs D2	*p* < 0.537
cD2 vs D2	*p* < 0.992

Data are given as mean (median, interquartile ranges). The surgeon’s categorical classification was retrospectively adjusted based on the VAS score. The following definitions were applied: D2 lymphadenectomy was classified as a VAS score ≤ 7.7, cD2 as VAS score > 7.7 to ≤ 8.0, CME as VAS score > 8.0 to ≤ 9.0, and D3 as VAS score > 9.0 (Fig. 1C)

^a^One-way ANOVA. Statistically significant at 5%

^b^Tukey’s post hoc test

**Fig. 3:**
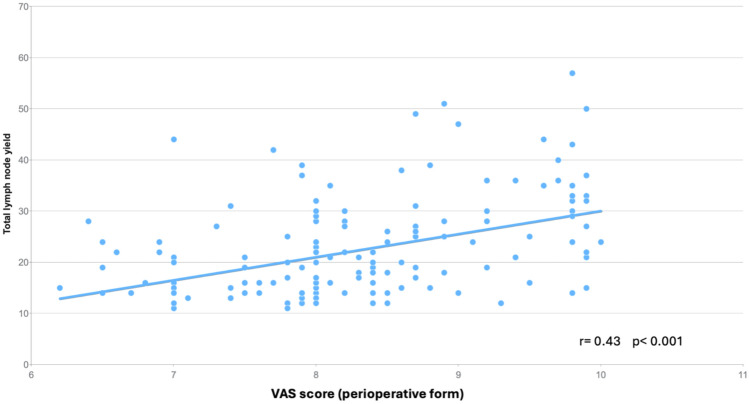
Relationship between VAS score and total number lymph nodes harvested analyzed with linear regression and Pearson’s correlation coefficient

The superior mesenteric vein was not visible in 24 patients (15%) and visible in 130 patients (84%). The superior mesenteric artery was not visible in 97 patients (62%) and visible in 57 (37%); the gastrocolic trunk of Henle was not visible in 75 patients (48%) and visible in 79 patients (51%). The vascular ligation was described to the right of the superior mesenteric vein in 93 patients (60%) and at the origin in 60 patients (39%). The specimen quality was described as type 0 (best) in 84 patients (54%), type 1 in 57 patients (37%), and type 2 in 9 patients (6%). The corresponding VAS scores differed significantly (Table 3). Linear regression confirmed a positive correlation between increasing VAS score and the number of harvested lymph nodes (*r* = 0.43, *p* < 0.001, Fig. 3).

**Table 3:** VAS score and correlation with anatomical landmarks, specimen quality, surgical access, and surgeon's experience

	Number (%)	VAS score	*p*-value
SMV visible			*p* < 0.001^a^
No	24 (15)	7.1 (7.0, 6.8–7.5)	
Yes	130 (84)	8.4 (8.4, 8.0–9.2)	
SMA visible			*p* < 0.001^a^
No	97 (62)	7.9 (8.0, 7.5–8.4)	
Yes	57 (37)	9.0 (9.2, 8.4–9.8)	
GTH visible			*p* < 0.001^a^
No	75 (48)	7.8 (7.9, 7.3–8.0)	
Yes	79 (51)	8.9 (8.7, 8.3–9.7	
MCA visible			*p* < 0.001^a^
No	30 (19)	7.7 (7.6, 7.0–8.0)	
Yes	124 (80)	8.5 (8.4, 8.0–9.2)	
Vascular ligation			*p* < 0.001^a^
To the right of SMV	93 (60)	7.8 (8.0, 7.4–8.1)	
Central	60 (39)	9.1 (9.2, 8.6–9.8)	
Specimen quality			*p* < 0.001^b^
Type 0	84 (54)	8.7 (8.6, 8.2–9.5)	
Type 1	57 (37)	7.8 (7.9 7.3–8.0)	
Type 2	9 (6)	7.2 (6.9, 6.5–7.9)	
Type 3	0 (0)	N/A	
Surgical access			*p* < 0.001^b^
Open	56 (36)	8.9 (9.3, 8.0–9.8)^c^	
Laparoscopic	49 (32)	7.9 (8.0, 7.7–8.3)^d^	
Robotic	50 (32)	8.0 (8.2, 7.8–8.4)^d^	
D3 surgeon			*p* < 0.001
No	28 (18)	7.5 (7.7, 7.0–8.0)	
Yes	127 (82)	8.5 (8.4, 8.0–9.2)	

Data are given as mean (median, interquartile ranges)

*SMV* superior mesenteric vein, *SMA* superior mesenteric artery, *GTH* gastrocolic trunk of Henle, specimen quality: Benz classification (8). D3 surgeon part of the operating team

^a^Mann-Whitney U-test

^b^One-way ANOVA. Statistically significant at 5%

^c^Tukey’s post hoc test. Significant difference open vs laparoscopic and open vs robotic access

^d^No significant difference between laparoscopic vs robotic access

Table 4 presents the comparison of lymphadenectomy categories based on the extent of mesenteric excision, evaluated using the VAS score. The data show that more extensive lymphadenectomy is associated with improved visibility of vascular key structures, enhanced specimen quality, and an increased yield of both positive and total lymph nodes. These findings suggest a direct correlation between the extent of mesenteric excision and surgical as well as pathological outcomes.

**Table 4:** Comparison of lymphadenectomy categories based on mesenteric excision according to the VAS score

Lymphadenectomy category	Dissection extent	Visiblevessels	Specimen quality	*N* status: positive nodes	Total lymph nodes retrieved
**D2** (VAS ≤ 7.7) *n* = 32	Central dissection to the right of SMV	***SMV visible*** 10(32) ***SMA visible*** 3(10) ***GTH visible*** 0(0) ***MCA visible*** 15(48)	*Type 0* 3(10) *Type 1* 23(74) *Type 2* 5(16)	0.6 (0, 0–1)	19 (16, 14–22)
**CME** (VAS > 7.7–9) *n* = 89	SMV exposure to the right of SMA	***SMV visible*** 86(96) ***SMA visible*** 22 (25) ***GTH visible*** 49(55) MCA visible 77(86)	*Type 0* 54(62) *Type 1* 29(33) *Type 2* 4(5)	1.7 (0, 0–2)	21 (18, 14–25)
**D3** (VAS > 9) *n* = 34	SMA exposure to the left of SMV	***SMV visible*** 34(100) ***SMA visible*** 32(94) ***GTH visible*** 30(88) ***MCA visible*** 32(94)	*Type 0* 27(84) *Type 1* 5(16) *Type 2* 0(0)	2.9 (0.5, 0–5.2)	30 (30,23–36)

Data are given as *n* (%) and mean (median, interquartile ranges)

*VAS* Visual analogue scale, *SMV* superior mesenteric vein, *SMA* superior mesenteric artery, *GTH* gastrocolic trunk of Henle, *MCA* middle colic artery, specimen quality: Benz classification (8)

## Discussion

The extent of lymphadenectomy in the present study showed considerable variation, with the planned extent not always being achieved. In 14% of patients, a planned D3 lymphadenectomy was not performed, likely because of intraoperative challenges such as obesity or complex vascular anatomy. Additionally, there was a significant discrepancy between the surgeon’s categorical classification and the classification based on the surgeon’s VAS score.

A complete D2 lymphadenectomy, defined by a VAS score between > 7.7 and ≤ 8.0, was correctly classified in 38 (46%) patients, while the surgeon's classification was used for 82 patients. Forty-eight patients had a VAS score between > 8.0 and ≤ 9.0, meeting the criteria for CME. The cD2 category represents a narrow range and was often misapplied because of the lack of an alternative classification. A D2 lymphadenectomy was incorrectly classified in three (10%) patients and a D3 lymphadenectomy in ten (23%) patients. These findings highlight the limitations of categorical classifications in accurately describing the extent of lymphadenectomy.

The VAS score offers the opportunity to update and refine categorical classifications based on the latest definitions (D2, cD2, CME, and D3). In this study, the total lymph node yield according to the corrected classification, based on the surgeon’s VAS score, revealed significant differences, with the highest yield observed in the D3 group.

The VAS score showed significant variation depending on the visibility of anatomical landmarks, including the superior mesenteric vein, superior mesenteric artery, gastrocolic trunk of Henle (GTH), middle colic artery, and the level of vascular ligation. This suggests that the VAS score reflects not only the extent of lymphadenectomy at the level of the ileocolic vessels but also centrally around the GTH.

The VAS score also discriminated significantly between the grades of specimen quality. Linear regression showed a positive correlation between the VAS score and the number of lymph nodes harvested.

A major limitation of the study is the self-reporting of VAS scores by the operating surgeon, which may introduce observer bias. However, internal consistency was assessed by comparing the reported scores with the documentation of visualized vascular landmarks. Future validation through independent observers and intraoperative imaging may enhance the objectivity and reproducibility of the VAS scoring system.

Surgeon-reported classifications were reassigned based on VAS scores, which introduces a risk of circular validation, as the classification and the score are not fully independent. While this represents a methodological limitation, the primary aim of the VAS was not validation but to provide a structured, quantitative description of mesenteric dissection. In this context, it may serve as a tool to support the ongoing refinement of anatomical classifications in line with evolving surgical practice.

Another limitation is the single-center design and the involvement of highly experienced colorectal surgeons, which may limit the generalizability of the findings to other clinical settings. Concurrently, a considerable number of less experienced surgeons contributed to the data. While four senior surgeons with established expertise in D3 lymphadenectomy participated in most procedures, the operations were also performed by 18 additional surgeons during their specialist training. This variation in surgical experience may have introduced heterogeneity in both technique and VAS scoring despite structured training and intraoperative supervision intended to ensure consistency.

Despite extensive experience with extended lymphadenectomy in the department, there was inconsistency in how surgeons understood and applied the categorical classifications, which also changed over time. The limited choice between D2, cD2, and D3 on the paper form made it challenging to accurately describe the dissection of the superior mesenteric vein (SMV).

A recent systematic review identified 6 surgical techniques and 35 definitions for extended lymphadenectomy for radical right-sided colectomy, demonstrating the great heterogeneity and consistent overlap among definitions. Central vascular ligation was the only step described in all studies [10].

A consensus statement on CME for right-sided cancer recommended central vascular ligation, exposure of the SMV, and excision of an intact mesocolon as the essential components [15]. The extent of lymphadenectomy for CME is not defined clearly and either described as dissection of the SMV [13] or exposure of the SMV alongside the SMA [16]. The Japanese D3 dissection is an overlapping concept to CME that involves the same surgical principals but is associated with a shorter bowel resection [17]. The Japanese D3 lymphadenectomy is based on two principles, the lymph node dissection to the root of the feeding artery and dissection according to lymph flow based on Gillot’s concept with dissection of the nodes of the “surgical trunk.” The term “surgical trunk” is limited to the lymphovascular tissue along the SMV between the ileocolic vein and the GTH, meaning the right ventrolateral SMV [18].

In the present study, the medial D3 border was defined as the left lateral margin of the SMA [12]. In the RELARC study, CME was defined as dissection of the central lymphatic tissue covering the surface of the SMA and SMV and the root of the middle colic artery. While this study found no evidence that CME was associated with superior disease-free survival outcomes compared to standard D2 lymph node dissection, a subgroup analysis revealed that among patients with stage III cancer, the DFS was nearly 10 percentage points higher in the CME group compared to the D2 group. The standard D2 dissection was defined as the division of the feeding vessel at the right edge of the superior mesenteric vein (SMV) [8].

The inconsistent use of terminology and variations in categorical classifications complicate the interpretation of outcome studies on the extent of lymphadenectomy.

The VAS score offers a reproducible, anatomy-based assessment of lymphadenectomy, independent of procedural definitions such as CME or D2–D3, which remain subject to interpretation and ongoing international consensus efforts. By referencing specific anatomical landmarks—such as the degree of SMV exposure—the VAS minimizes subjectivity and allows for consistent intraoperative documentation. While high vessel ligation is central to lymph node yield in CME, the VAS complements this by providing a precise and gradable measure of dissection extent, applicable across varying levels of surgical experience.

The VAS is simple to apply, directly linked to intraoperative anatomy, and correlates with central dissection around key vascular structures such as the gastrocolic trunk of Henle and the middle colic artery—particularly relevant for tumors at the hepatic flexure. Its integration into surgical registries would allow for standardized documentation of lymphadenectomy and support large-scale analyses to define optimal oncologic resection for right-sided colon cancer.

## Supplementary Information

Below is the link to the electronic supplementary material.

Supplementary file1 (PDF 8615 KB)

Supplementary file2 (JPG 1404 KB)

Supplementary file3 (PDF 205 KB)

## Data Availability

No datasets were generated or analysed during the current study.
