# Supplementary material for: Proposal of a new visual analogue scale to describe the extent of lymphadenectomy in right-sided colectomy for cancer—a prospective observational study
Source: Tech Coloproctol. 2025 Sep 2;29(1):166. doi: 10.1007/s10151-025-03182-8 (PMC12405331; doi:10.1007/s10151-025-03182-8)

Right colectomy: Intraoperative form 1

Operation date: ___________
Surgeon: ___________
Patient name/ study ID: ___________
Birthdate: ___________

Anatomy preop CT:
- ICA in front of SMA: Yes ☐ No ☐ NA ☐
- JJ veins in front of SMA: Yes ☐ No ☐ NA ☐
- Distance of ICV to GTH (mm) ___________

Planned extend of resection:
- D2 ☐
- Complete D2 ☐
- D3 ☐

Access:
- Robotic ☐
- Laparoscopic ☐
- Open ☐
- Converted ☐

Anastomosis:
- Extracorporeal ☐
- Intracorporeal ☐
- Handsewn ☐
- Stapler ☐

Operation performed:
- D2 ☐
- Complete D2 ☐
- D3 ☐

Bleeding:
- None ☐
- Surgical hemostasis ☐
- Blood loss (ml) ___________

Intraoperative surgical complications:
- None ☐
- Bowel lesion ☐
- Vascular lesion ☐
- Other ☐

Intraoperative general complications:
- None ☐
- Cardiac ☐
- Respiratory ☐
- Other ☐

Specimen quality:

0 ☐

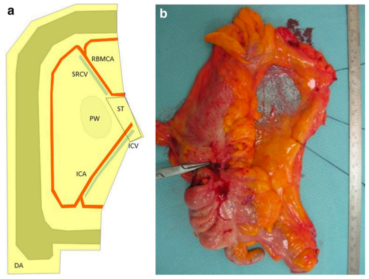

I ☐

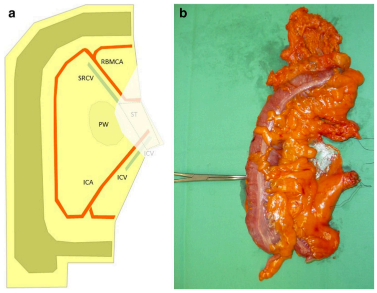

II ☐

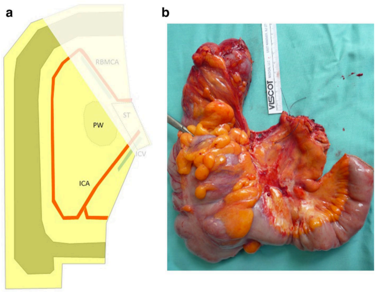

III ☐

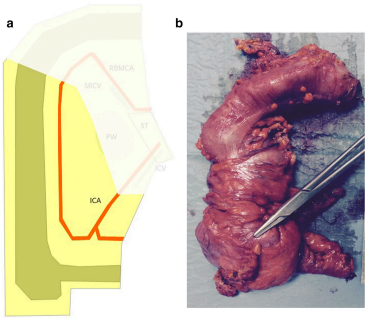

Right colectomy: Intraoperative form 2

Vascular anatomy:

Blood vessel visualized:

- SMV ☐
- GTH ☐
- SMA ☐
- MCA ☐

Level of vascular ligature:

- To the right of SMV ☐
- Central ☐

Photodocumentation: ☐

Extend of lymphadenectomy:

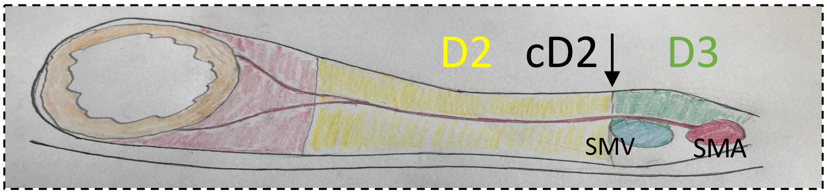

Mark with cross

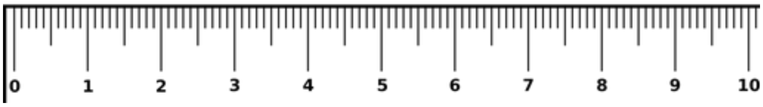

Supplement: Supplementary file 1 — Supplementary file1 (PDF 8615 KB) [file 10151_2025_3182_MOESM1_ESM.pdf]
