# Supplementary figures and images for: Proposal of a new visual analogue scale to describe the extent of lymphadenectomy in right-sided colectomy for cancer—a prospective observational study
Source: Tech Coloproctol. 2025 Sep 2;29(1):166. doi: 10.1007/s10151-025-03182-8 (PMC12405331; doi:10.1007/s10151-025-03182-8)

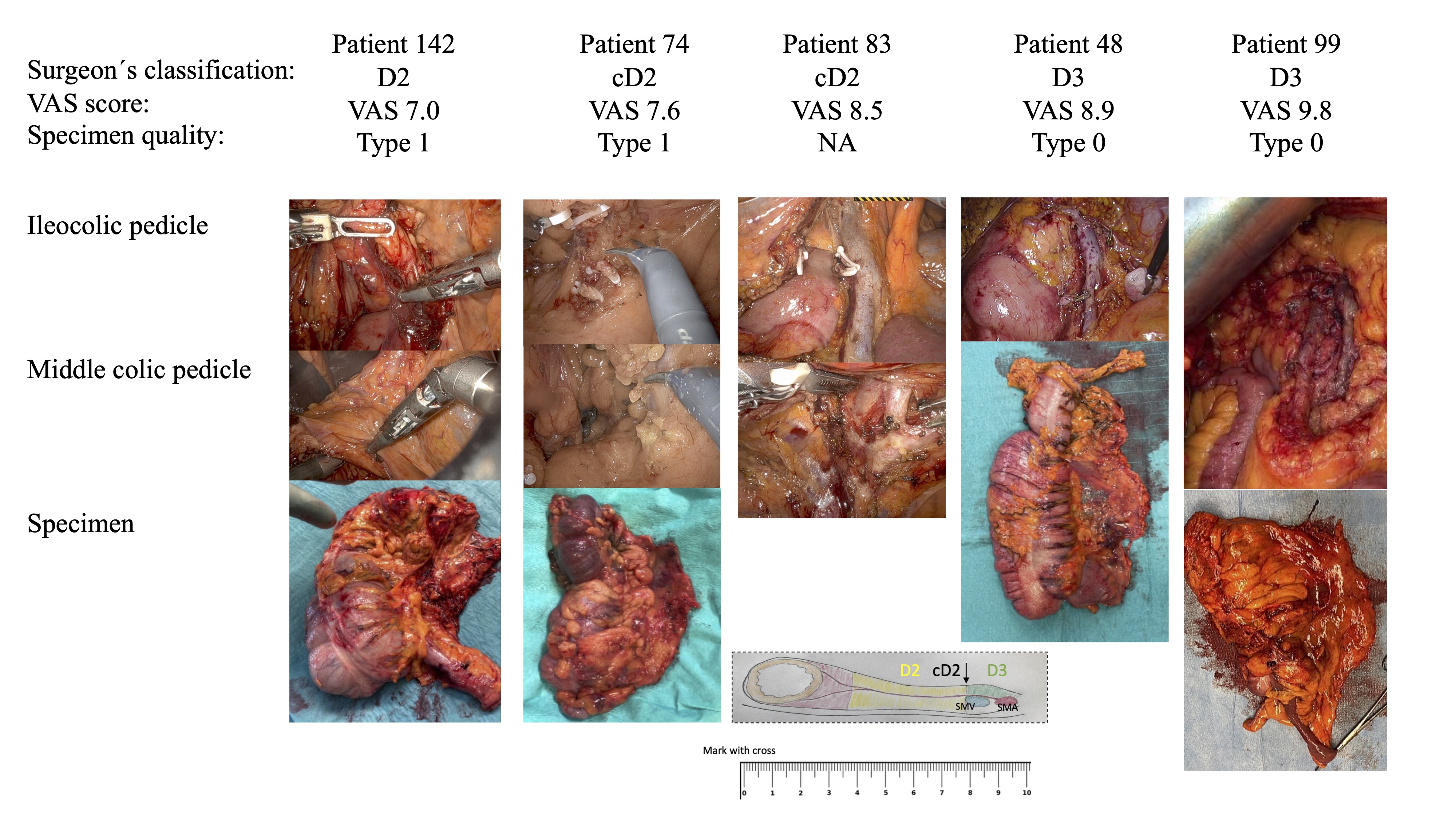

Supplement: Supplementary file 2 — Supplementary file2 (JPG 1404 KB) [file 10151_2025_3182_MOESM2_ESM.jpg]
