# Supplementary material for: Proposal of a new visual analogue scale to describe the extent of lymphadenectomy in right-sided colectomy for cancer—a prospective observational study
Source: Tech Coloproctol. 2025 Sep 2;29(1):166. doi: 10.1007/s10151-025-03182-8 (PMC12405331; doi:10.1007/s10151-025-03182-8)
Supplement: Supplementary file 3 — Supplementary file3 (PDF 205 KB) [file 10151_2025_3182_MOESM3_ESM.pdf]

Legends to figures:

Figure 1: A: Preoperative computed tomography (CT) with contrast, slice thickness 1 mm and three-dimensional volume rendering Osirix reconstruction of the root of the midgut mesentery. B: Same patient as in A: Image acquired at surgery. C: Schematic drawing of the mesenteric vessels superior mesenteric vein (SMV) and superior mesenteric artery (SMA) at the level of the ileocolic vessels combined with a visual analogue scale. Categorical lymph node dissection: D2, complete D2 (cD2), complete mesocolic excision (CME) and D3. Red arrow represents the extent of lymphadenectomy as shown in B. VAS score 9.8. Lymph node (LyN0), ileocolic vein (ICV), gastrocolic Trunk of Henle (GTH), middle colic artery (MCA), right colic artery (RCA), superior mesenteric vein (SMV) and superior mesenteric artery (SMA).

Figure 2: Density plot to illustrate the distribution of the VAS score and the total number of lymph nodes harvested. A: All patients. B: Patients grouped after the surgeon`s categorical classification.

Figure 3: Relationship between VAS score and total number lymph nodes harvested analyzed with linear regression and Pearson´s correlation coefficient.

Figure S1. Example of the intraoperative documentation form used to assess the extent of lymphadenectomy:

The form includes the Visual Analogue Scale (VAS) score (Fig. 1), documentation of mesenteric vessel visualization, and both the planned and achieved categorical extent of lymphadenectomy. Additional data recorded include blood loss, surgical access method, and specimen quality. The categorical extent of lymphadenectomy (D2, cD2, and D3) was assigned in accordance with national guidelines (9). Specimen quality was evaluated using the Benz classification system (13).

Figure S2. Additional patient examples illustrating the application of the Visual Analogue Scale (VAS):

- **Patient 142**: Robotic-assisted operation. The ileocolic pedicle was dissected at an intermediate level, and the transverse colon was divided without visualization of vascular structures. Classified as D2 with a VAS score of 7.0.

Specimen quality: Type 1 (re-evaluated as Type 2 based on the image). Pathology: pT2N0 (0/14).

- **Patient 74**: Robotic-assisted operation. The ileocolic vessels were divided at their origin, but the SMV was not clearly visible due to obesity. The right branch of the middle colic artery (RBMCA) was divided. Classified as cD2 with a VAS score of 7.6. Specimen quality: Type 1. Pathology: pT3N0 (0/16).

- **Patient 83**: Robotic-assisted operation. The SMV, MCA, and RBMCA were completely visualized. Classified as cD2 with a VAS score of 8.5. Specimen quality: Not assessed (NA). Pathology: pT3N0 (0/22).

- **Patient 48**: Laparoscopic extended right-sided colectomy. The SMV and gastrocolic trunk of Henle (GTH) were completely visualized, and the MCA was divided at its origin. Classified as D3 with a VAS score of 9.0. Specimen quality: Type 0. Pathology: pT4aN1 (3/51).

- **Patient 99**: Open operation. The SMV and SMA were completely visualized. Classified as D3 with a VAS score of 9.8. Specimen quality: Type 0. Pathology: pT3N1 (2/29).

This figure demonstrates the variability in surgical technique, anatomical visualization, and pathological outcomes as assessed by the VAS scoring system.
